# Supplementary material for: Stacking Model‐Based Classifiers for Dealing With Multiple Sets of Noisy Labels
Source: Biom J. 2025 Mar 12;67(2):e70042. doi: 10.1002/bimj.70042 (PMC11898607; doi:10.1002/bimj.70042)
Supplement: Supplementary file 1 — Supporting Information [file BIMJ-67-e70042-s001.zip › density-based-ensemble-model-main/competitors_real_data/netlab-master/netlab-master/help/olgd.htm]

Netlab Reference Manual olgd


# olgd

## Purpose

On-line gradient descent optimization.

## Description

`[net, options, errlog, pointlog] = olgd(net, options, x, t)` uses
on-line gradient descent to find a local minimum of the error function for the
network
`net` computed on the input data `x` and target values
`t`. A log of the error values
after each cycle is (optionally) returned in `errlog`, and a log
of the points visited is (optionally) returned in `pointlog`.
Because the gradient is computed on-line (i.e. after each pattern)
this can be quite inefficient in Matlab.

The error function value at final weight vector is returned
in `options(8)`.

The optional parameters have the following interpretations.

`options(1)` is set to 1 to display error values; also logs error
values in the return argument `errlog`, and the points visited
in the return argument `pointslog`. If `options(1)` is set to 0,
then only warning messages are displayed. If `options(1)` is -1,
then nothing is displayed.

`options(2)` is the precision required for the value
of `x` at the solution. If the absolute difference between
the values of `x` between two successive steps is less than
`options(2)`, then this condition is satisfied.

`options(3)` is the precision required of the objective
function at the solution. If the absolute difference between the
error functions between two successive steps is less than
`options(3)`, then this condition is satisfied.
Both this and the previous condition must be
satisfied for termination. Note that testing the function value at each
iteration roughly halves the speed of the algorithm.

`options(5)` determines whether the patterns are sampled randomly
with replacement. If it is 0 (the default), then patterns are sampled
in order.

`options(6)` determines if the learning rate decays. If it is 1
then the learning rate decays at a rate of `1/t`. If it is 0
(the default) then the learning rate is constant.

`options(9)` should be set to 1 to check the user defined gradient
function.

`options(10)` returns the total number of function evaluations (including
those in any line searches).

`options(11)` returns the total number of gradient evaluations.

`options(14)` is the maximum number of iterations (passes through
the complete pattern set); default 100.

`options(17)` is the momentum; default 0.5.

`options(18)` is the learning rate; default 0.01.

## Examples

The following example performs on-line gradient descent on an MLP with
random sampling from the pattern set.

```
net = mlp(5, 3, 1, 'linear');
options = foptions;
options(18) = 0.01;
options(5) = 1;
net = olgd(net, options, x, t);
```

## See Also

`graddesc`

---

**Pages:**
Index


---

Copyright (c) Ian T Nabney (1996-9)
